# Cell Line-Based Human Bladder Organoids with Bladder-like Self-Organization—A New Standardized Approach in Bladder Cancer Research

**DOI:** 10.3390/biomedicines11112958

**Published:** 2023-11-01

**Authors:** Mandy Berndt-Paetz, Shanfu Han, Annett Weimann, Annabell Reinhold, Sandra Nürnberger, Jochen Neuhaus

**Affiliations:** 1 Department of Urology, Research Laboratories, Leipzig University, 04103 Leipzig, Germany; annett.weimann@medizin.uni-leipzig.de (A.W.); annabell.reinhold@medizin.uni-leipzig.de (A.R.); sandra.nuernberger@medizin.uni-leipzig.de (S.N.); 2 Clinical Apartment, Cornerstone MedTech (Beijing) Limited, Beijing 100005, China; lishi8090@cueb.edu.cn

**Keywords:** bladder cancer cell lines, organoids, self-organization, stratification, Wnt/β-catenin activation, drug response

## Abstract

Three-dimensional tumor models have gained significant importance in bladder cancer (BCa) research. Organoids consisting of different cell types better mimic solid tumors in terms of 3D architecture, proliferation, cell–cell interaction and drug responses. We developed four organoids from human BCa cell lines with fibroblasts and smooth muscle cells of the bladder, aiming to find models for BCa research. The organoids were characterized in terms of cytokeratins, vimentin, α-actin and KI67 by immunoreactivity. Further, we studied ligand-dependent activation of the Wnt/β-catenin pathway and investigated the responses to anti-tumor therapies. The organoids mimicked the structure of an inverse bladder wall, with outside urothelial cells and a core of supportive cells. The cytokeratin staining patterns and proliferation rate were in conjunction with the origins of the BCa cells. RT-112 even showed stratification of the epithelium. Treatment with Wnt10B led to increased β-catenin (active) levels in high-grade organoids, but not in low-grade BCa cells. Doxorubicin treatment resulted in clearly reduced viability (10–30% vs. untreated). In contrast, the effectivity of radiotherapy depended on the proliferation status of BCa cells. In conclusion, cell-line-based organoids can form bladder-like structures and reproduce in vivo features such as urothelial differentiation and stratification. Thus, they can be useful tools for functional studies in BCa and anti-cancer drug development.

## 1. Introduction

Bladder cancer (BCa) is the eleventh-most prevalent cancer in the world (Globocan 2020; https://gco.iarc.fr/, accessed on 6 July 2023). Approximately 70% of patients have non-muscle-invasive disease (stage Ta, T1, CIS) at diagnosis, and 30% have muscle-invasive urothelial carcinomas. The standard therapy for non-muscle-invasive bladder cancer (NMIBC) includes transurethral resection of the bladder, followed by adjuvant chemo-or immunotherapy [1]. For muscle-invasive bladder cancer (MIBC), a radical cystectomy with or without adjuvant chemotherapy is recommended [2]. Despite optimal management, recurrences of NMIBC are common, and there are no organ-preserving therapy options for MIBC. The lack of well-characterized culture models for the malignant bladder epithelium hampers the development of new therapies.

Recently, several studies have shown the value of 3D tumor cultures (spheroids and organoids) for modeling various aspects of tumor biology. Organoids are the most often used 3D cell cultures that combine different cell types to mimic the natural histological structures of organs [3,4]. Tumor organoids reproduce many features of in vivo tumors, including 3D architecture, oxygen and nutrient gradients, different proliferation rates, complex cell–cell interactions, extracellular matrix deposition and chemoresistance [3,5]. Both cell line-based and patient-derived organoid models have been successfully established for BCa research. For example, more than 70 patient-derived xenografts for urothelial carcinoma from lower urinary tract malignancies have been described [6,7]. Patient-derived BCa organoids (PDOs) have also been successfully established from resection samples ranging from NMIBC to MIBC. Using immunohistochemistry and sequence analysis, it has been shown that these resulting BCa organoids contained both basal and luminal tumor cell subtypes. PDOs from individual BCa patients retained their genetic aberrations and reproduced the histopathological phenotype of the original tumor in vitro [8,9,10,11]. Therefore, PDOs have been used for studying BCa progression, for evaluating patient-specific drug responses and for advancing novel treatment strategies [10,11]. However, the reported success rates in the establishment of tissue-derived BCa organoids vary between 30–100%, mainly in terms of the number of obtained organoids, suitability for drug testing, long-term survival and successful serial passaging [12].

In addition to primary patient-derived models, various cell-line-based organoids and spheroids have been developed. Most 3D models from commercial BCa cell lines have been generated with the use of an artificial extracellular matrix. For example, BCa cell lines cultured in Matrigel (RT-4, RT-112, EJ) were shown to form cell–matrix interactions that affect gene expression and may be important for BCa progression [13]. In another study, human BCa cells (HTB-9; ATCC 5637) were cultured in a bioreactor with a rotating vessel preventing cell attachment. The generated 3D cultures expressed specific urothelial differentiation markers and allowed for analyses of interactions between uropathogenic *E. coli* and the human urothelium [14]. In contrast, Goulet et al. used tissue engineering to develop a multilayered stroma equivalent consisting of multiple cell layers of an endothelium/fibroblast mixed culture and a urothelial cell layer, in which they integrated spheroids from RT-4 and T-24 BCa cells [15].

Even though cell lines are susceptible to genetic changes and loss of tumor heterogeneity during immortalization, cell-line-derived organoids are characterized by high viability and standardization. Therefore, organoids of a range of well-established BCa cell lines representative of the spectrum of the disease (NMIBC vs. MIBC; G1-4) can be used as standardized and reproducible models for assessing treatment responses, especially for therapies acting in a mutation- and resistance-independent manner (e.g., radiotherapy). In this study, we used the ultra-low attachment microplate method to develop a urinary bladder organoid model consisting of bladder carcinoma cells (BCa cell lines; RT-4, RT-112, T-24, CAL-29; G1-4), primary human bladder fibroblasts and primary bladder smooth muscle cells. The organoids (Orgs) were characterized according to their morphologies and bladder-like structures, urothelial differentiation and proliferation. Further, we analyzed ligand-dependent activation of the cancer-associated Wnt signaling pathway and investigated the responses to anti-tumor therapies.

## 2. Materials and Methods

### 2.1. Cell Culture

Four human BCa cell lines of various origins (Table 1) were obtained from the Leibniz Institute DSMZ (German collection of microorganisms and cell cultures, GmbH, Braunschweig, Germany).

Primary human bladder fibroblasts (hBFs, FC-0050) and human bladder smooth muscle cells (hBSMCs, FC-0043) were both purchased from CellSystems (Troisdorf, Germany). All BCa cell lines were cultured in RPMI1640 medium supplemented with 10% FBS (Biochrom, Berlin, Germany) and 1% CTS™ GlutaMAX™-I Supplement (Thermo Fisher Scientific, Dreieich, Germany). The hBFs were cultured in FibroLife^®^ S2 medium (LL-0011), and the hBSMCs were cultured in VascuLife^®^ SMC medium (LL-0014; Lifeline Cell Technology, Frederick, MD, USA). The cells were maintained at 37 °C and 5% CO_2_ in a humidified atmosphere. To obtain a single-cell suspension of each cell line, Accutase cell detachment solution (PanBiotech, Aidenbach, Germany) was applied.

### 2.2. Generation of Standardized BCa Organoids

The ultra-low attachment (ULA) microplate method [16] was used for organoid formation. To generate BCa organoids, BCa cells were mixed with hBF and hBSMC at equal densities (7000 cells each). The cell suspensions (21,000 cells per well) were centrifuged (250× *g*; 5 min) into Nunclon™ Sphera™ 96-well ULA round-bottom plates (Thermo Fisher Scientific, Dreieich, Germany). Self-assembling organoids were cultured in a 1:1:1 mixture of cell-type-specific media for 4 days until histology and DAB immunostaining for morphological characterization were carried out. For drug and radiation responses, cell suspensions (12,000 cells per well; 4000 cells each) were seeded into Corning^®^ Spheroid microplates (black; #4520; Corning Inc., Kennebunk, ME, USA). BCa Orgs were freshly prepared for each experiment and kept for a maximum of 1 week in culture.

### 2.3. Wnt Ligand Treatment

The recombinant Wnt10B peptide was purchased from R&D Systems (Minneapolis, MN, USA). Wnt10B stock solution (100 µg/mL) was prepared in PBS containing 0.1% bovine serum albumin (PBS/0.1% BSA). BCa cells (2D), grown on collagen A-coated (PC-3) 13 mm coverslips, were treated with 500 ng/mL Wnt10B, which was diluted in culture medium. BCa Orgs, cultured in ULA plates, were treated with 1000 ng/mL Wnt10B. After the indicated time periods, at 37 °C, samples were assayed for canonical Wnt pathway activation (non-phospho (active) β-catenin) by immunofluorescence. Samples treated with PBS/0.1% BSA and diluted in culture medium served as vehicle-treated controls.

### 2.4. Histology and DAB Immunostaining

Organoids were fixed using 4% paraformaldehyde and embedded into HistoGel (Thermo Fisher Scientific, Dreieich, Germany). Serial sections at 4 µm were HE-stained and immunostained following paraffin embedding. For DAB staining, sections were deparaffinized followed by antigen retrieval using heat-induced epitope retrieval buffer (pH 9.0; Zytomed Systems, Berlin, Germany) for 40 min at 100 °C. After blocking of the endogenous peroxidase, the sections were treated with primary antibodies (Table 2) overnight at 4 °C.

Biotin-linked secondary antibodies (goat-anti-rabbit, goat-anti-mouse; Vector Laboratories, Newark, NJ, USA) were incubated for 45 min at room temperature. Following a coupling step with horseradish peroxidase streptavidin, DAB was used for visualization and the nuclei were counterstained with hematoxylin. Images were acquired using a Keyence BZ-X800 microscope (Keyence Coorporation, Osaka, Japan). An analysis of DAB stains was conducted using the IHC profiler plugin [17] in Fiji, including ImageJ version 1.53 [18].

### 2.5. Immunofluorescence

Cells (2D) were fixed with ice-cold methanol and permeabilized using a DMSO/Triton-X-100 mixture. Paraffin sections of BCa Orgs were permeabilized as described above. Primary antibodies against panCK (Table 2) and β-catenin (non-phospho (active) b-catenin (Ser33/37/Thr41), D13A1, Cell Signaling Technology, Frankfurt/Main, Germany) was incubated overnight at 4 °C. Alexa Fluor 555^®^/Alexa Fluor488^®^-coupled secondary antibodies (Thermo Fisher Scientific, Dreieich, Germany) diluted in TBS (1 h, room temperature) were used for indirect immunofluorescence. Nuclei in the 2D-cultured cells were labeled with 4′,6-Diamidine-2′-phenylindole dihydrochloride (DAPI, Cat.-No.: D9542, Sigma-Aldrich, Munich, Germany). Nuclei in the BCa Orgs were visualized with TO-PRO-3 iodide (Thermo Fisher Scientific, Dreieich, Germany). The samples were analyzed via confocal laser scanning microscopy (LSM 800, Carl Zeiss, Jena, Germany). The fluorescence intensities (ex/em: 553/568 nm) of the images were quantified using ImageJ [18].

### 2.6. Drug and Radiation Response

Prior to treatment, BCa cells (2D) were cultured for 24 h and BCa Orgs were cultured for 96 h in 96-well plates. The samples were treated with doxorubicin (0, 0.1, 0.5, 1.0, 2.5, 3.5, 5.0, 7.5, 10.0 µM; diluted in culture medium). To assess the radiation response, the samples were treated with ionizing radiation (0, 2, 4, 6, 9, 12 Gy) using a 150 kV orthovoltage X-ray machine (Xstrahl 200, Xstrahl, Ratingen, Germany). Cytotoxic effects in 2D-cultured BCa cells were analyzed with the CellTiter-Blue^®^ Cell Viability Assay (ex/em: 560/590), and the cytotoxicity of BCa Orgs was assayed using a CellTiter-Glo^®^ 3D Cell Viability Assay after 72 h. Both assay types were obtained from Promega (Mannheim, Germany) and measured on a SpectraMax M5 microplate reader (Molecular Devices, Sunnyvale, CA, USA).

### 2.7. Statistical Analysis

Statistical analyses were performed using Prism 9.0 software (GraphPad Software Inc., La Jolla, SD, USA). Bar diagrams present the means + standard deviations from at least three independent experiments. The Gaussian distribution of values was tested using the D’Agostino–Pearson omnibus normality test. Statistical differences were analyzed by one-way ANOVA and the Mann–Whitney test. The Pearson correlation coefficient (r) and the coefficient of determination (r^2^) were calculated for the correlation analysis. Drug response values and IC50 were calculated by applying nonlinear regression (curve fit; inhibitor vs. response).

## 3. Results

Urinary bladder organoids from four BCa cell lines were established. The BCa organoids (BCa Orgs) were characterized according to their morphological structure and marker expression. Further, responses to Wnt ligand stimulation as well as anti-tumor treatments were analyzed.

### 3.1. Morphological Characterization of BCa Orgs

**Figure 1:**
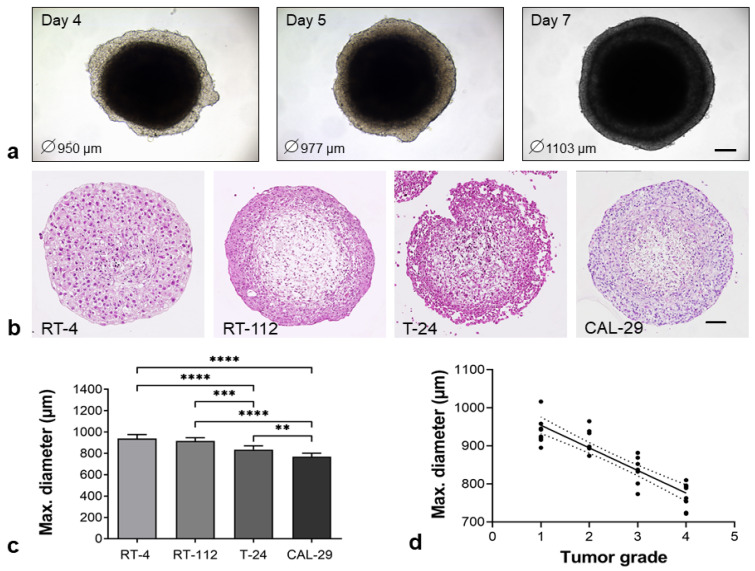
Spontaneous formation of heterogeneous, compact BCa organoids. BCa cells (RT-4, RT-112, T-24, CAL-29) were co-cultured with hBF and hBSMC in an ULA plate. (**a**) Development of organoids over a period of up to 7 days. Live cell observation by transmitted light using a LSM800, exemplarily shown for RT-112 organoids. Spheroids reached sizes of up to 1 mm in diameter after 1 week. (**b**) Morphology of human BCa Orgs by HE stains after 4 days in culture. The morphology of BCa Orgs varied depending on the BCa cell line utilized (scale bar: 100 µm). (**c**) Organoid sizes ranged from 650 to 1000 µm in diameter, ** *p* < 0.01, *** *p* < 0.001, **** *p* < 0.0001. One-way ANOVA, mean + SD, *n* = 8. (**d**) Correlation analysis. Diameter of Orgs negatively correlated with the grading of BCa cell lines, Pearson r^2^ = 0.7875, *p* < 0.0001.

BCa cells were co-cultured with hBF and hBSMC in 96-well ULA plates. Spontaneous formation of uniformly shaped spheroids was observed within 24 h and continued until 7 days after seeding; this was exemplarily shown for RT-112 organoids on days 4, 5 and 7 (Figure 1a). An overview on organoid formation from day 1 to 4 is shown in Figure S1. The organoids were fixed, histologically processed and morphologically characterized by HE-overview staining.

After 4 days, all organoids showed separate structures, with peripheral cell layers and inner cores. While the RT-4, RT-112 and CAL-29 organoids formed a tightly packed periphery, T-24 organoids showed a loose arrangement of cells in the outer layer (Figure 1b). The organoids reached a maximum diameter of 650–1000 µM after 96 h, showing no signs of central necrosis. The diameters of the BCa Orgs decreased with the increasing grading of the cell lines which were used (Figure 1c,d). Except for RT-4, all BCa Orgs lost their compact structures after 7 days of culture (Figure S1b). Therefore, 4-day-old organoids were used for characterization.

#### 3.1.1. Bladder-like Self-Organization

**Figure 2:**
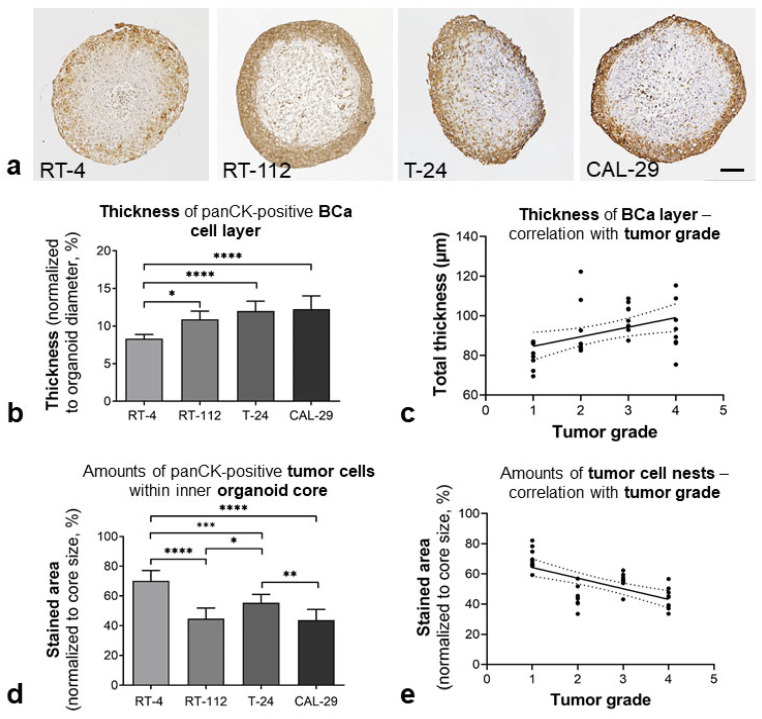
Bladder-like self-organization of BCa organoids. Distribution of BCa cells in urinary bladder organoids by analysis of panCK immunoreactivity (IR). (**a**) Representative images of BCa Orgs immunostained for panCK (brown); cell nuclei (blue). BCa cells showed different levels of cell separation, with the formation of an urothelium-like peripheral cell layer and tumor cell nests inside the organoid core. Scale bar: 100 µm. (**b**) Thickness of the BCa cell layer, normalized to the organoid size. The thickness of the RT-4 cell layer (RT-4: papillary, low-grade) was significantly lower than in BCa Orgs consisting of high-grade tumor cells, * *p* < 0.05, **** *p* < 0.0001. One-way ANOVA, mean + SD, *n* = 8. (**c**) There was a significant correlation of BCa layer thickness with the grading of the BCa cells, Pearson r^2^ = 0.1879, *p* < 0.0132. (**d**) Quantification of panCK-positive BCa cell nests in the inner core; stained area (%) was determined. RT-4 Orgs showed significantly higher amounts of panCK-positive cell nests within the inner cores than high-grade BCa Orgs, * *p* < 0.05, ** *p* < 0.01, *** *p* < 0.001, **** *p* < 0.0001. One-way ANOVA, mean + SD, *n* = 8. (**e**) The panCK-IR in the inner core was negatively correlated with the grading of the cell line which was used. Pearson r^2^ = 0.3686, *p* < 0.0002.

**Figure 3:**
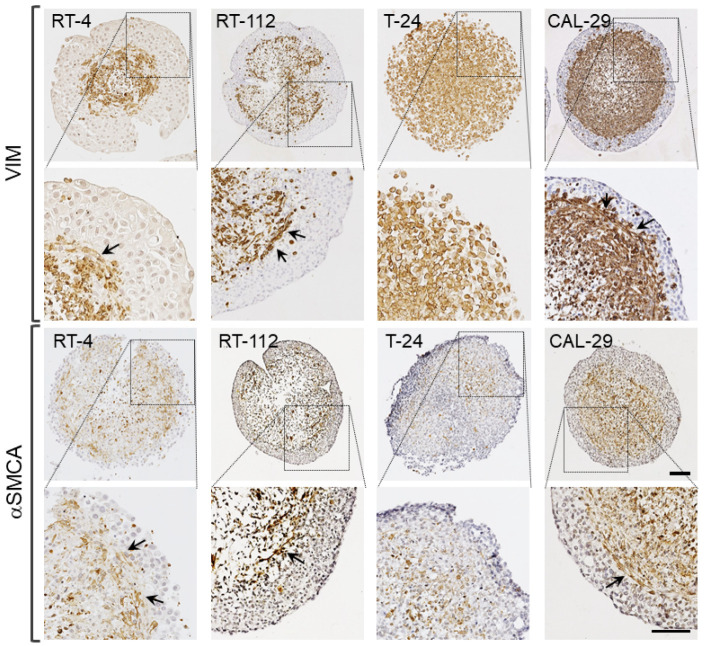
Bladder-like self-organization of BCa organoids. Distribution of hBF and hBSMC in urinary bladder organoids by analysis of VIM and αSMCA immunoreactivity. Representative images of BCa Orgs immunostained for VIM and αSMCA (brown), cell nuclei (blue). VIM- and αSMCA-IR were complementary to panCK staining and confirmed the presence of hBF and hBSMC in the organoid core. Arrows indicate the formation of VIM- and αSMCA-positive elongated cells underlying the urothelium. Scale bar: 100 µm.

The distribution of cell types within organoids was examined by means of immunohistological staining against cytokeratins (panCK: BCa), vimentin (VIM: hBF) and alpha smooth muscle cell actin (αSMCA: hBSMC). Staining further evidenced extensive separation of the cell types (Figure 2 and Figure 3).

The cells showed an organotypic self-organization, with the formation of panCK-positive luminal epithelial layers and inner cores consisting of supportive VIM/αSMCA-positive cells, as well as panCK-positive BCa cell nests (Figure 2a and Figure 3). Further, RT-4, RT-112 and CAL-29 Orgs showed an abundance of VIM- and αSMCA-positive elongated cells underlying the urothelium (arrows, Figure 3). In this regard, the BCa cells showed a varying tendency to form a urothelium-like luminal epithelium. While RT-4, RT-112 and CAL-29 formed a clearly discernible epithelial layer, T-24 showed an irregular surface without the formation of a typical urothelium, as well as numerous intra-spheroidal BCa nests (Figure 2a).

For further characterization, the thickness of the peripheral tumor cell layer and the number of panCK-positive cells in the spheroid core were determined. The average layer thickness ranged from approximately 80 µm in RT-4 organoids to 90 µm in the RT-112, T-24 and CAL-29 organoids The BCa layer thickness, normalized to the organoid diameter, ranged from approximately 8% in RT-4 organoids to 12% in T-24/CAL-29 organoids and correlated with the BCa cell grading (Figure 2b,c). In addition, the amount of BCa cell nests was determined by analyzing the panCK-positive area in the inner spheroid core. Here, low-grade RT-4 organoids showed significantly more BCa cell nests than the RT-112, T-24 and CAL-29 organoids. Among high-grade BCa Orgs, the cores of T-24 organoids bore the most panCK-positive BCa cell nests. The amounts of intra-spheroidal BCa cells negatively correlated with the grading of the utilized BCa cell lines. In addition, the high immunoreactivity of the tight junction-associated protein claudin 4 (CL4) was demonstrated, while the ZO-1 immunoreactivity was very low (Figure S2).

#### 3.1.2. Urothelial Differentiation

**Figure 4:**
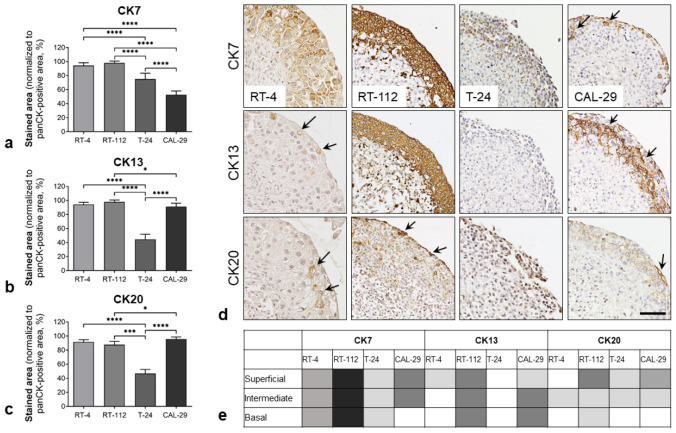
Urothelial differentiation of BCa cells in organoids. Analysis of different CKs to detect poorly (CK7, basal), moderately (CK13, intermediate) or well-differentiated (CK20, superficial, umbrella cells) epithelial cells in BCa Orgs. (**a**–**c**) Quantification of CK7 (**a**), CK13 (**b**) and CK20 (**c**) immunoreactivity in the BCa cell layer: * *p* < 0.05, *** *p* < 0.001, **** *p* < 0.0001. One-way ANOVA, mean + SD, *n* = 8. (**d**,**e**) Stratification of the urothelial-like peripheral cell layer. Distribution of CK7-, CK13- and CK20-positive cells in BCa organoids. (**d**) Immunohistochemical staining of CK7, CK13 and CK20 in the peripheral cell layer. Arrows refer to the labeling of superficial cells and the staining of intermediate and basal cells, respectively. Scale bar: 100 µm. (**e**) Schematic overview of CK staining patterns in BCa Orgs.

The cytokeratin expression patterns can provide valuable information regarding the differentiation statuses of epithelial cells. The IR of CK7 (poorly differentiated), CK13 (moderately differentiated) and CK20 (well-differentiated) was examined in the tumor cell layers of BCa Orgs (Figure 4).

T-24 organoids exhibited significantly lower levels of CK7-, CK13- and 20-positive areas within the BCa layer. RT-4 and RT-112 showed nearly equal amounts of CKs within the panCK-positive tumor cell area, while CAL-29 Orgs showed higher CK13-and CK20-immunoreactivity compared to CK7 (Figure 4a–c). Interestingly, the BCa Orgs showed different distributions of the CKs within the individual layers (basal, intermediate and superficial) (Figure 4d,e). RT-112 organoids showed uniform CK7 and CK13 immunoreactivity in all cell layers, whereas CK20 was highly expressed in the superficial cell layer (Figure 4d). RT-4 also showed uniform CK7 immunoreactivity in all BCa cell layers; CK13 and CK20 were only abundant in intermediate and superficial cells. CAL-29 exhibited CK7 staining of superficial and intermediate cells and CK13 staining of intermediate and basal cells; CK20 was weakly expressed by intermediate and superficial cells (Figure 4d). T-24 showed no urothelial-typical CK expression profile in the organoid and, thus, no evidence of stratification of the epithelium (Figure 4d,e). Uroplakin-II and Uroplakin-III were analyzed in order to characterize urothelial plaque assembly as a marker of a terminally differentiated urothelium. Both markers were negative in all tested BCa organoids (Figure S3).

#### 3.1.3. Proliferation Status

**Figure 5:**
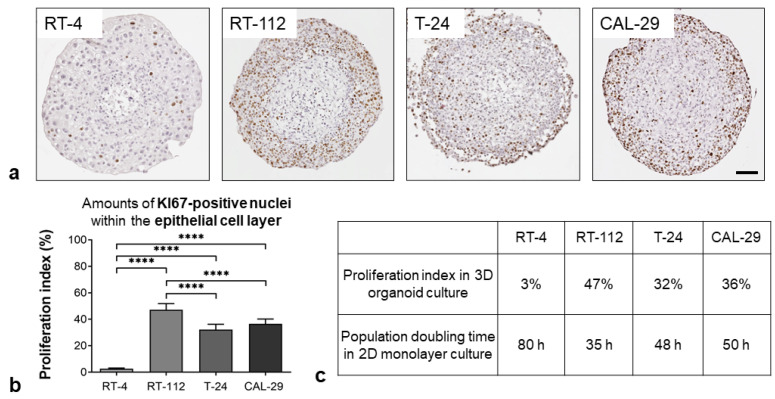
Proliferation status. Analysis of KI67 immunoreactivity in BCa cells. (**a**) Representative images of BCa Orgs immunostained for KI67 (brown), cell nuclei (blue). Scale bar: 100 µm. (**b**) Quantification of KI67-positive nuclei indicated a vigorous proliferation of tumor cells in the RT-112, T-24 and CAL-29 organoids, whereas low-grade RT-4 Orgs showed a proliferation index of <10%; **** *p* < 0.0001. One-way ANOVA, mean + SD, *n* = 8. (**c**) Proliferation indices were in conjunction with appropriate population doubling times of BCa cells in monolayer cultures.

KI67 served as a marker for the proliferation of cells within the tumor cell layers of BCa organoids (Figure 5).

The staining of KI67 evidenced proliferative activity of RT-112, T-24 and CAL-29 BCa cells (Figure 5a). The proliferation index ranged from approximately 35% in T-24 and CAL-29 organoids to 50% in RT-112 organoids, while RT-4 organoids showed almost no proliferative activity (Figure 5b). Therefore, the proliferation indices were in conjunction with the appropriate population doubling times of BCa cells in monolayer cultures (Figure 5c). The hBF/hBSMC core showed no labeling in the organoids.

### 3.2. Ligand-Dependent Wnt Pathway Activation

**Figure 6:**
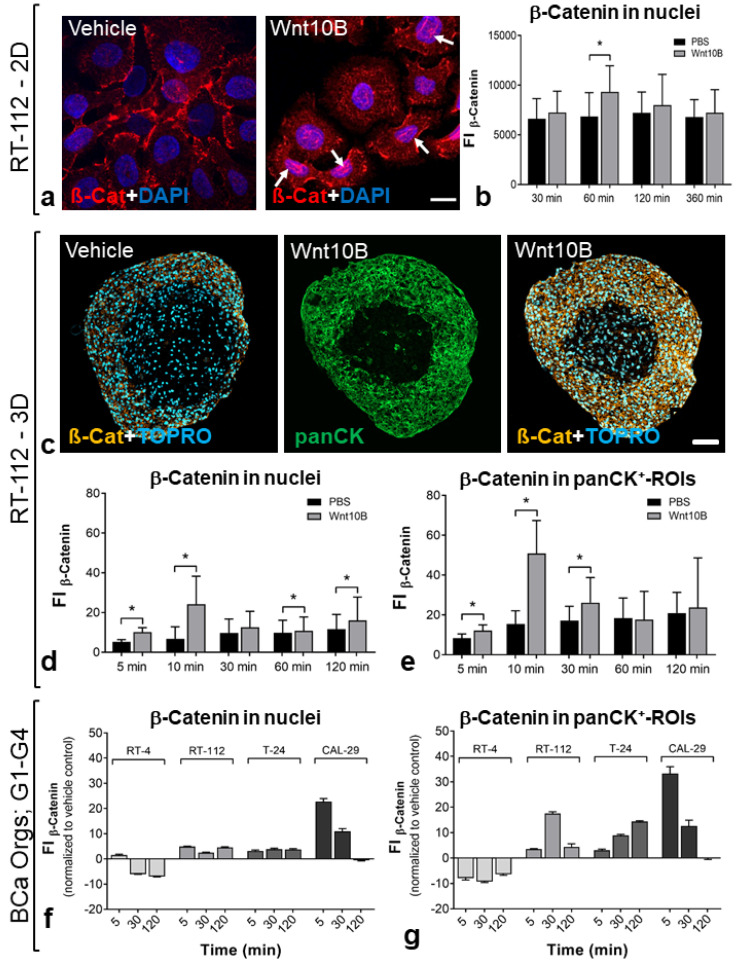
Activation of the Wnt/β-catenin pathway in human BCa organoids by Wnt10B. (**a**,**b**) Translocation of active β-catenin into the nucleus in 2D cultured RT-112 cells. (**a**) Immunofluorescence staining of β-catenin (non-phospho, Ser33/37/Thr41). Membrane association was observed in control cells (vehicle control); Wnt10B-treated cells showed nuclear localization of β-catenin; arrows indicate nuclear β-catenin labeling. β-catenin (red), nuclei (blue). Scale bar: 75 µm. (**b**) Quantification of active β-catenin (FI; fluorescence intensity) in DAPI-positive nuclei revealed significantly increased nuclear β-catenin levels after 30 min of Wnt10B vs. controls. * Significance vs. vehicle-treated control (*p* ≤ 0.05, Mann–Whitney test), mean + SD. (**c**–**g**) Increase in active β-catenin in the tumor cell layers of 3D cultured BCa cells. (**c**) Immunofluorescence staining of β-catenin (non-phospho, Ser33/37/Thr41) in paraffin-embedded BCa organoids, examplarily shown for RT-112 organoids. Localization of β-catenin was observed mainly in the submembranous cytoskeleton, but also in the nuclei of RT-112 tumor cells. β-catenin (orange), panCK (green), nuclei (blue). Scale bar: 75 µm. (**d**) Quantification of active β-catenin (FI; fluorescence intensity) in panCK-positive ROIs revealed significantly increased β-catenin levels after 5, 10 and 30 min of Wnt10B (vs. controls) in RT-112; (**e**) nuclear β-catenin levels were increased after 5, 10, 30 and 120 min. * Significance vs. vehicle-treated control (*p* ≤ 0.05, Mann–Whitney test), mean + SD. (**f**,**g**) Increased β-catenin (FI; fluorescence intensity; normalized to PBS vehicle control) was also detected in the BCa layer of T-24 and CAL-29 organoids (especially in panCK-positive ROIs), while RT-4 cells showed no response to Wnt10B.

Induction of the Wnt/β-catenin pathway was provoked by treatment with Wnt10B, a Wnt ligand associated with canonical Wnt signaling [19]. The cytoplasmic and nuclear translocation of non-phospho (active) β-catenin was analyzed using immunofluorescence to examine the downstream activation of Wnt (Figure 6).

Wnt10B incubation resulted in obvious cytoplasmic and nuclear accumulation of β-catenin in 2D-cultured RT-112 cells (Figure 6a). The fluorescence intensity of β-catenin staining was quantified in DAPI-positive nuclei (Figure 6b). Significantly increased nuclear β-catenin was detected after 60 min of Wnt10B incubation.

In BCa Orgs, the increase in active β-catenin was restricted to the tumor cell layer. Further, localization of β-catenin was observed mainly in the submembranous cytoskeleton, but also in the nuclei of BCa cells (exemplarily shown for RT-112; Figure 6c). Quantification of active β-catenin (FI; fluorescence intensity) in panCK-positive ROIs and TOPRO-positive nuclei revealed a significantly increased cytoplasmic/nuclear β-catenin ratio already after 5 to 10 min of Wnt10B (vs. controls) in RT-112 (Figure 6d). Increased β-catenin was also detected in the BCa layers of T-24 and CAL-29 organoids (especially in panCK-positive ROIs), while RT-4 cells showed no response to Wnt10B. In this regard, CAL-29 cells showed the most rapid response to Wnt10B stimulation (Figure 6f,g).

### 3.3. Response to Anti-Tumor Therapies

**Figure 7:**
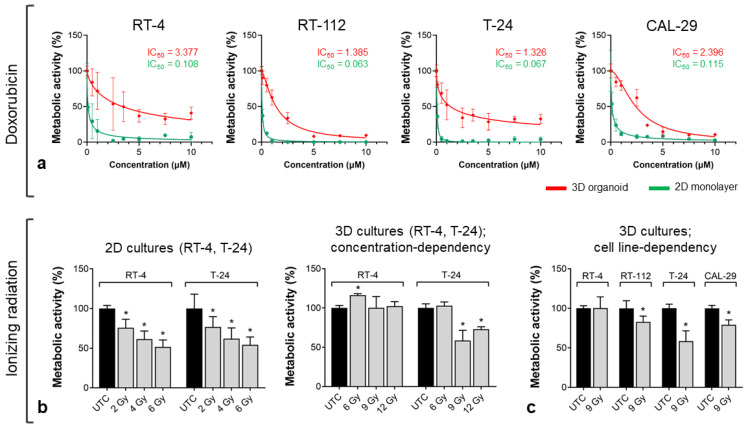
Response to anticancer treatment. Metabolic activity was determined using the CellTiter-Glo^®^ 3D Cell Viability Assay in BCa Orgs and the CellTiter-Blue^®^ Cell Viability Assay in BCa monolayer cultures 72 h after treatment. (**a**) Drug response curves for BCa Orgs and monolayers treated with doxorubicin. IC50 values were determined by nonlinear regression of the dose response (inhibitor vs. normalized response). (**b**) Dose-dependent cytotoxic effects of radiotherapy on RT-4 and T-24 Orgs compared to monolayer cultures. * Significance vs. untreated control (UTC), *p* < 0.05, one-way ANOVA, mean + SD, *n* = 3. (**c**) Cell-line-dependent effects of radiotherapy in BCa Orgs. * Significance vs. untreated control (UTC), *p* < 0.05, Mann–Whitney test, mean + SD, *n* = 3.

To explore the utility of BCa organoids as preclinical models for the evaluation of anti-cancer therapies, we performed treatment response assays in 3D BCa Orgs versus conventional monolayer cell cultures. Anticancer therapies were selected based on their clinical relevance for bladder cancer treatment, including standard-of-care chemotherapy (doxorubicin) and radiotherapy being performed in individual non-operable patients (Figure 7).

The organoids were subjected to a range of concentrations of doxorubicin and incubated for 72 h. The cytotoxic effects were determined by quantification of the metabolic activity. We observed differences in the responses of BCa Orgs to drug treatment. While BCa cells in the monolayer cultures showed an almost complete attenuation of their metabolic activity, the decrease was much less in the organoids. In RT-112 and CAL-29 organoids, doxorubicin treatment resulted in a decrease in the metabolic activity of approximately 90%, while RT-4 and T-24 organoids showed a reduction of approximately 70% versus untreated controls. In addition, in monolayers, the IC50 values were 20- to 30-fold lower than in Orgs (20-fold: RT-112, T-24, CAL-29; 30-fold: RT-4) (Figure 7a).

The dose-dependent cytotoxicity of radiotherapy was tested in RT-4 and T-24 cells. Radiotherapy using 9 Gy resulted in significant cytotoxic effects in T-24, while RT-4 Orgs were not affected by IR. Interestingly, the corresponding 2D cultures of RT-4 and T-24 exhibited similar responses to IR independently of their proliferation status (Figure 7b). In BCa Orgs, the treatment efficacy of IR strongly depended on the proliferation index of BCa cells (3% in RT-4 vs. 30–45% in RT-112/T-24/CAL-29) within the organoid cultures (Figure 7c).

## 4. Discussion

In the past decade, 3D models have gained much importance in BCa research. The available model systems provide a valuable complement to standard cell culture methods. In our study, hetero-typed BCa organoids consisting of several BCa cells representing the entire range of malignancy grades were generated without the use of any artificial ECM. We co-cultured human BCa cells, human primary bladder fibroblasts and bladder smooth muscle cells in round-bottomed wells of ULA microplates, which resulted in the spontaneous formation of single, uniformly sized BCa organoids in each well within 24 h post-seeding.

Our urinary bladder organoids exhibited an organotypic self-organization, with the formation of a urothelial-like peripheral cell layer surrounding the spheroid core upon contact with the cell culture medium. Furthermore, we were able to show that this peripheral cell layer partly presents barrier properties (CL-4 expression). The ability of urinary bladder cells to self-assemble in vitro has already been demonstrated in previous studies. Ingram and colleagues used a bioreactor with a slowly rotating chamber (6 rpm, constant free-fall) to create standardized organoids consisting of fibroblasts (HBL-13) covered with BCa cells (HBL-2 cell line) [20]. Kim et al. generated so-called “assembloids“ by co-culturing representative patient-derived BCa organoids (luminal, basal) with the corresponding primary tumor-associated fibroblasts and endothelial cells [21]. In addition to the peripheral epithelial layer, isolated BCa cell nests remained in the spheroids during generation. This appears to be advantageous, especially with regard to the development of focal therapies (e.g., radiotherapy, photodynamic therapy), as it allows for testing of the treatment’s ablative effects. The BCa organoid types (RT-4, RT-112, T-24 and CAL-29) differed significantly in their properties. BCa cells showed a varying tendency to form a urothelium-like luminal epithelium, the layer thickness of which correlated with the respective grading of the BCa cell line. The epithelial layers of BCa organoids also showed significant differences in cytokeratin expression patterns as markers of the differentiation statuses of the cells. Thus, the RT-4 and RT-112 organoids showed considerable expression of CK7, CK13 and CK20, whereas the T-24 cells showed significantly lower immunoreactivity of these markers. Corresponding expression patterns for the RT-4, RT-112 and T-24 monolayer cultures have already been described in the literature [22,23]. Moreover, we observed that the distributions of CK7, CK13 and CK20 differed in the epithelial layers (basal, intermediate and superficial). In particular, RT-112 showed evidence of a stratified urothelium with CK7- and CK13-positive basal and intermediate cells and CK20-positive superficial cells. The ability of RT-112 to stratify has previously been demonstrated in co-cultures of RT-112 and stroma cells [23]. However, BCa Orgs showed no formation of uroplakin plaques, indicating failure to reach terminal urothelial differentiation. Significant differences were also observed when analyzing the proliferation status of BCa cells in the organoid. While RT-4 showed almost no proliferative activity, RT-112, T-24 and CAL-29 exhibited brisk proliferation. The proliferation index correlated with the respective doubling times in conventional monolayer cultures. The results of spheroid characterization demonstrated that the essential properties of BCa cells (e.g., marker expression, CK profile, proliferative potential) were preserved in the organoids. Thus, standardized cell-line-based BCa organoids seem to be suitable for the investigation of specific therapeutic effects in different bladder carcinomas.

In this regard, the suitability of BCa Orgs as preclinical models for the evaluation of anticancer therapies was tested by analyzing the treatment response to doxorubicin and radiotherapy. Although doxorubicin was reported to have poor penetrance beyond the first three or four cell layers within organoids [24], chemotherapeutic treatment resulted in clearly reduced organoid viability (10–30% vs. UTC), especially in the well-layered RT-112 and CAL-29 organoids. The IC50 values ranged from 1.3 in RT-112 and T-24 to 3.4 in RT-4 organoids, which is in accordance with previous studies showing similar IC50 values in doxorubicin-treated PDOs [8]. The BCa monolayer cultures exhibited even higher cytotoxicity (2% viability vs. UTC) to doxorubicin. Reduced chemotherapeutic sensitivity in 3D cultures was recently shown in cisplatin-treated urinary tract PDOs in comparison to the corresponding 2D cultures [25]. The effectiveness of radiotherapy using ionizing radiation depended on the proliferation status of BCa cells in organoids. While proliferative inactive RT-4 was not affected by IR, T-24 organoids showed cytotoxic effects, reaching a 40% reduction in viability. In contrast, the 2D cultures showed similar responses to IR independently of their proliferative potential. These findings further support the view that 2D cultured cells show modified cellular behavior (e.g., proliferation), and that the phenotypes of different BCa cell types are better represented in 3D cultures.

There are comparatively few studies on the role of the Wnt signaling pathway in urinary bladder carcinoma. Nevertheless, β-catenin is significantly overexpressed in urinary bladder tumors, and the expression correlates with tumor stage, tumor cell differentiation and poor prognosis [26]. Furthermore, it has been shown that the signaling pathway is also activated in BCa cell lines [27]. To explore whether the Wnt pathway in BCa Orgs was functional, we provoked Wnt pathway activation by the canonical Wnt ligand Wnt10B and subsequently quantified the downstream effector non-phospho (active) β-catenin. In BCa organoids, activation of the Wnt/β-catenin pathway was restricted to tumor cells in the RT-112, T-24 and CAL-29 organoids, which showed significantly enhanced cytoplasmic and nuclear β-catenin after only 5 to 10 min of Wnt10B. Compared to 2D-cultured BCa cells, the increase in active β-catenin was substantially higher in the BCa organoids. Interestingly, high-grade CAL-29 cells exhibited the most rapid β-catenin (active) increase, while low-grade RT-4 showed no Wnt10B response. This is in line with the results of Yoshida and colleagues demonstrating the cytoplasmatic and nuclear translocation of β-catenin in organoids of the BCa cell lines that were treated with the Wnt/β-catenin activator CHIR99021. They found that Wnt/β-catenin activation increased the proliferation and viability of BCa cells in organoids, but not in conventional adherent cultures [28]. Responses to Wnt pathway activation varied across different BCa models, which underscores the importance of organoid cultures for drug screening in vitro.

Many other organoid models have been successfully established for BCa research. The available model systems include porous scaffolds for mimicking a tissue-like microenvironment, 3D bioprinting and Organ(oid)-on-a-chip [29]. For instance, so-called bioprinters have been developed to enable the computer-assisted design and printing of 3D cancer models or whole organs, which allows for control of the cell distribution within the 3D structure [30]. In the field of BCa research, bioprinting has been used to fabricate a 3D organoid consisting of BCa cells imprinted on a base of vascular endothelial cells and lung fibroblasts. The organoids were placed in a special microfluidic chamber allowing for separate fluid flow (basally, medially and apically) and treated with BCG (Bacillus Calmette-Guérin). They found reduced cell viability, modulation of inflammatory cytokines and targeted migration of co-cultured monocytes (THP-1) into the organoids. The described technology enables a controlled supply of oxygen and nutrients to lead to the formation of complex multicellular cultures [31]. However, the application of 3D bioprinting and Organ(oid)/Cancer-on-a-chip experiments in high-throughput analyses is currently in its infancy. Compared with other organoid generation techniques (e.g., hanging drop, bioreactor, Organ(oid)-on-a-chip), the ULA microplate method is simple, convenient and less time-consuming. This method also allows for co-cultures of different cell types, including cancer cells and stromal cells, without the influence of artificial ECM components. The system is suitable for high-throughput drug screening using microplate readers or confocal analysis systems [29]. The option to gradually expand this culture system, e.g., by the inclusion of immune cells to investigate basic immunological processes, is another exciting prospect.

## 5. Conclusions

In summary, our model can be used to produce a large number of standardized and reproducible organoid samples suitable for functional studies of BCa progression and pharmacological screenings. Most important is the finding that there are significant differences in the effects of anti-tumor therapies on 2D cultures and 3D organoid cultures. On the one hand, this suggests an effect of direct or indirect cell–cell interactions in a complex organotypic 3D culture, and on the other hand, it might reflect limitations in terms of the accessibility of drugs into cells. Therefore, organoid cultures are predicted to improve and help in the development of BCa therapies. An exiting field is the application of focal anticancer therapies causing not only direct tumor destruction, but also immune stimulation. Thus, a co-culture of immune cells and BCa organoids could be a useful approach to evaluate new immunogenic treatment options and/or immunotherapies.

## Figures and Tables

**Table 1:** Characteristics of the bladder cancer cell lines utilized in this study. Acc.-No., accession number (DSMZ); MIBC, muscle-invasive bladder cancer; NMIBC, non-muscle-invasive bladder cancer.

Cell Line	Acc.-No.	Origin (Gender, Age, Stage)	Invasiveness	Grade	Subtype
RT-4	ACC-412	male, 63 y, T2	NMIBC	1	luminal
RT-112	ACC-418	female, n.n., n.n.	NMIBC	2	luminal
T-24	ACC-376	female, 81 y, n.n.	MIBC	3	mixed
CAL-29	ACC-515	female, 80 y, T2	MIBC	4	luminal

**Table 2:** List of primary antibodies used for organoid characterization. Cat-No., catalog number; Ms, mouse; Rb, rabbit.

Antigen		Specificity	Host	Source	Cat.-No.	Dilution
Cytokeratin Pan	panCK	Epithelial cells	Ms	Sigma-Aldrich, Munich, Germany	C2931	1:400
Vimentin	VIM	VIM-positive cells, e.g., fibroblasts	Ms	Sigma-Aldrich, Munich, Germany	V6389	1:400
Alpha Smooth muscle cell actin	αSMCA	αSMCA-positive cells, e.g., detrusor myocytes	Ms	Sigma-Aldrich, Munich, Germany	A2547	1:1000
Cytokeratin 7	CK7	Poorly differentiated epithelial cells	Ms	Abcam, Cambridge, UK	ab9021	1:400
Cytokeratin 13	CK13	Moderately differentiated epithelial cells	Ms	Abcam, Cambridge, UK	ab101001	1:50
Cytokeratin 20	CK20	Well-differentiated epithelial cells	Rb	Abcam, Cambridge, UK	Ab53120	1:100
Uroplakin-II	UPL-II	Terminally differentiated urothelial cells	Rb	Novus Biologicals, Abingdon, UK	NBP2-33389	1:50
Uroplakin-III	UPL-III	Terminally differentiated urothelial cells	Ms	Abcam, Cambridge, UK	ab78196	1:30
KI67		Nuclei of proliferating cells	Ms	Dako, Glostrup, Denmark	M0722	1:50
Claudin 4	CL4	Tight junction protein CL4	Rb	Abcam, Cambridge, UK	ab15104	1:400
Zonula occludens-1	ZO-1	Tight junction protein ZO-1	Ms	BD Biosciences, Franklin Lakes, NJ, USA	610966	1:200

## Data Availability

The data presented in this study are available upon request from the corresponding author.

## Supplementary Materials

The following supporting information can be downloaded at: https://www.mdpi.com/article/10.3390/biomedicines11112958/s1, Figure S1. Live cell observation and histology of BCa organoids. Figure S2. Formation of cell–cell contacts in BCa organoids. Figure S3. Formation of uroplakin plaques in BCa organoids.

## References

[1] Babjuk M., Burger M., Capoun O., Cohen D., Compérat E.M., Dominguez Escrig J.L., Gontero P., Liedberg F., Masson-Lecomte A., Mostafid A.H. (2022). European Association of Urology Guidelines on Non-muscle-invasive Bladder Cancer (Ta, T1, and Carcinoma in Situ). Eur. Urol..

[2] Witjes J.A., Bruins H.M., Cathomas R., Compérat E.M., Cowan N.C., Gakis G., Hernández V., Linares Espinós E., Lorch A., Neuzillet Y. (2021). European Association of Urology Guidelines on Muscle-invasive and Metastatic Bladder Cancer: Summary of the 2020 Guidelines. Eur. Urol..

[3] Drost J., Clevers H. (2018). Organoids in cancer research. Nat. Rev. Cancer.

[4] Schutgens F., Clevers H. (2020). Human Organoids: Tools for Understanding Biology and Treating Diseases. Annu. Rev. Pathol..

[5] Doctor A., Seifert V., Ullrich M., Hauser S., Pietzsch J. (2020). Three-Dimensional Cell Culture Systems in Radiopharmaceutical Cancer Research. Cancers.

[6] Wei L., Chintala S., Ciamporcero E., Ramakrishnan S., Elbanna M., Wang J., Hu Q., Glenn S.T., Murakami M., Liu L. (2016). Genomic profiling is predictive of response to cisplatin treatment but not to PI3K inhibition in bladder cancer patient-derived xenografts. Oncotarget.

[7] Inoue T., Terada N., Kobayashi T., Ogawa O. (2017). Patient-derived xenografts as in vivo models for research in urological malignancies. Nat. Rev. Urol..

[8] Lee S.H., Hu W., Matulay J.T., Silva M.V., Owczarek T.B., Kim K., Chua C.W., Barlow L.J., Kandoth C., Williams A.B. (2018). Tumor Evolution and Drug Response in Patient-Derived Organoid Models of Bladder Cancer. Cell.

[9] Mullenders J., de Jongh E., Brousali A., Roosen M., Blom J.P.A., Begthel H., Korving J., Jonges T., Kranenburg O., Meijer R. (2019). Mouse and human urothelial cancer organoids: A tool for bladder cancer research. Proc. Natl. Acad. Sci. USA.

[10] Yu L., Li Z., Mei H., Li W., Chen D., Liu L., Zhang Z., Sun Y., Song F., Chen W. (2021). Patient-derived organoids of bladder cancer recapitulate antigen expression profiles and serve as a personal evaluation model for CAR-T cells in vitro. Clin. Transl. Immunol..

[11] Minoli M., Cantore T., Hanhart D., Kiener M., Fedrizzi T., La Manna F., Karkampouna S., Chouvardas P., Genitsch V., Rodriguez-Calero A. (2023). Bladder cancer organoids as a functional system to model different disease stages and therapy response. Nat. Commun..

[12] Medle B., Sjödahl G., Eriksson P., Liedberg F., Höglund M., Bernardo C. (2022). Patient-Derived Bladder Cancer Organoid Models in Tumor Biology and Drug Testing: A Systematic Review. Cancers.

[13] Smith B.A., Kennedy W.J., Harnden P., Selby P.J., Trejdosiewicz L.K., Southgate J. (2001). Identification of genes involved in human urothelial cell-matrix interactions: Implications for the progression pathways of malignant urothelium. Cancer Res..

[14] Smith Y.C., Grande K.K., Rasmussen S.B., O’Brien A.D. (2006). Novel three-dimensional organoid model for evaluation of the interaction of uropathogenic *Escherichia coli* with terminally differentiated human urothelial cells. Infect. Immun..

[15] Ringuette Goulet C., Bernard G., Chabaud S., Couture A., Langlois A., Neveu B., Pouliot F., Bolduc S. (2017). Tissue-engineered human 3D model of bladder cancer for invasion study and drug discovery. Biomaterials.

[16] Amaral R., Zimmermann M., Ma A.-H., Zhang H., Swiech K., Pan C.-X. (2020). A Simple Three-Dimensional In Vitro Culture Mimicking the In Vivo-Like Cell Behavior of Bladder Patient-Derived Xenograft Models. Cancers.

[17] Varghese F., Bukhari A.B., Malhotra R., De A. (2014). IHC Profiler: An open source plugin for the quantitative evaluation and automated scoring of immunohistochemistry images of human tissue samples. PLoS ONE.

[18] Schindelin J., Arganda-Carreras I., Frise E., Kaynig V., Longair M., Pietzsch T., Preibisch S., Rueden C., Saalfeld S., Schmid B. (2012). Fiji: An open-source platform for biological-image analysis. Nat. Methods.

[19] Wend P., Wend K., Krum S.A., Miranda-Carboni G.A. (2012). The role of WNT10B in physiology and disease. Acta Physiol..

[20] Ingram M., Techy G.B., Ward B.R., Imam S.A., Atkinson R., Ho H., Taylor C.R. (2010). Tissue engineered tumor models. Biotech. Histochem..

[21] Kim E., Choi S., Kang B., Kong J., Kim Y., Yoon W.H., Lee H.-R., Kim S., Kim H.-M., Lee H. (2020). Creation of bladder assembloids mimicking tissue regeneration and cancer. Nature.

[22] Moll R., Achtstätter T., Becht E., Balcarova-Ständer J., Ittensohn M., Franke W.W. (1988). Cytokeratins in normal and malignant transitional epithelium. Maintenance of expression of urothelial differentiation features in transitional cell carcinomas and bladder carcinoma cell culture lines. Am. J. Pathol..

[23] Booth C., Harnden P., Trejdosiewicz L.K., Scriven S., Selby P.J., Southgate J. (1997). Stromal and vascular invasion in an human in vitro bladder cancer model. Lab. Investig..

[24] Mehta G., Hsiao A.Y., Ingram M., Luker G.D., Takayama S. (2012). Opportunities and challenges for use of tumor spheroids as models to test drug delivery and efficacy. J. Control. Release.

[25] Wei Y., Amend B., Todenhöfer T., Lipke N., Aicher W.K., Fend F., Stenzl A., Harland N. (2022). Urinary Tract Tumor Organoids Reveal Eminent Differences in Drug Sensitivities When Compared to 2-Dimensional Culture Systems. Int. J. Mol. Sci..

[26] Wu G., Weng W., Xia P., Yan S., Zhong C., Xie L., Xie Y., Fan G. (2021). Wnt signalling pathway in bladder cancer. Cell. Signal..

[27] Schmid S.C., Sathe A., Guerth F., Seitz A.-K., Heck M.M., Maurer T., Schwarzenböck S.M., Krause B.J., Schulz W.A., Stoehr R. (2017). Wntless promotes bladder cancer growth and acts synergistically as a molecular target in combination with cisplatin. Urol. Oncol..

[28] Yoshida T., Sopko N.A., Kates M., Liu X., Joice G., McConkey D.J., Bivalacqua T.J. (2018). Three-dimensional organoid culture reveals involvement of Wnt/β-catenin pathway in proliferation of bladder cancer cells. Oncotarget.

[29] Neuhaus J., Rabien A., Reinhold A., Köhler L., Berndt-Paetz M. (2023). 3D Tumor Models in Urology. Int. J. Mol. Sci..

[30] Kim J., Jang J., Cho D.-W. (2021). Recapitulating the Cancer Microenvironment Using Bioprinting Technology for Precision Medicine. Micromachines.

[31] Kim J.H., Lee S., Kang S.J., Choi Y.W., Choi S.Y., Park J.Y., Chang I.H. (2021). Establishment of Three-Dimensional Bioprinted Bladder Cancer-on-a-Chip with a Microfluidic System Using Bacillus Calmette-Guérin. Int. J. Mol. Sci..

