# Supplementary material for: Cell Line-Based Human Bladder Organoids with Bladder-like Self-Organization—A New Standardized Approach in Bladder Cancer Research
Source: Biomedicines. 2023 Nov 1;11(11):2958. doi: 10.3390/biomedicines11112958 (PMC10669858; doi:10.3390/biomedicines11112958)
Supplement: Supplementary file 1 [file biomedicines-11-02958-s001.zip › Figure S1.pdf]

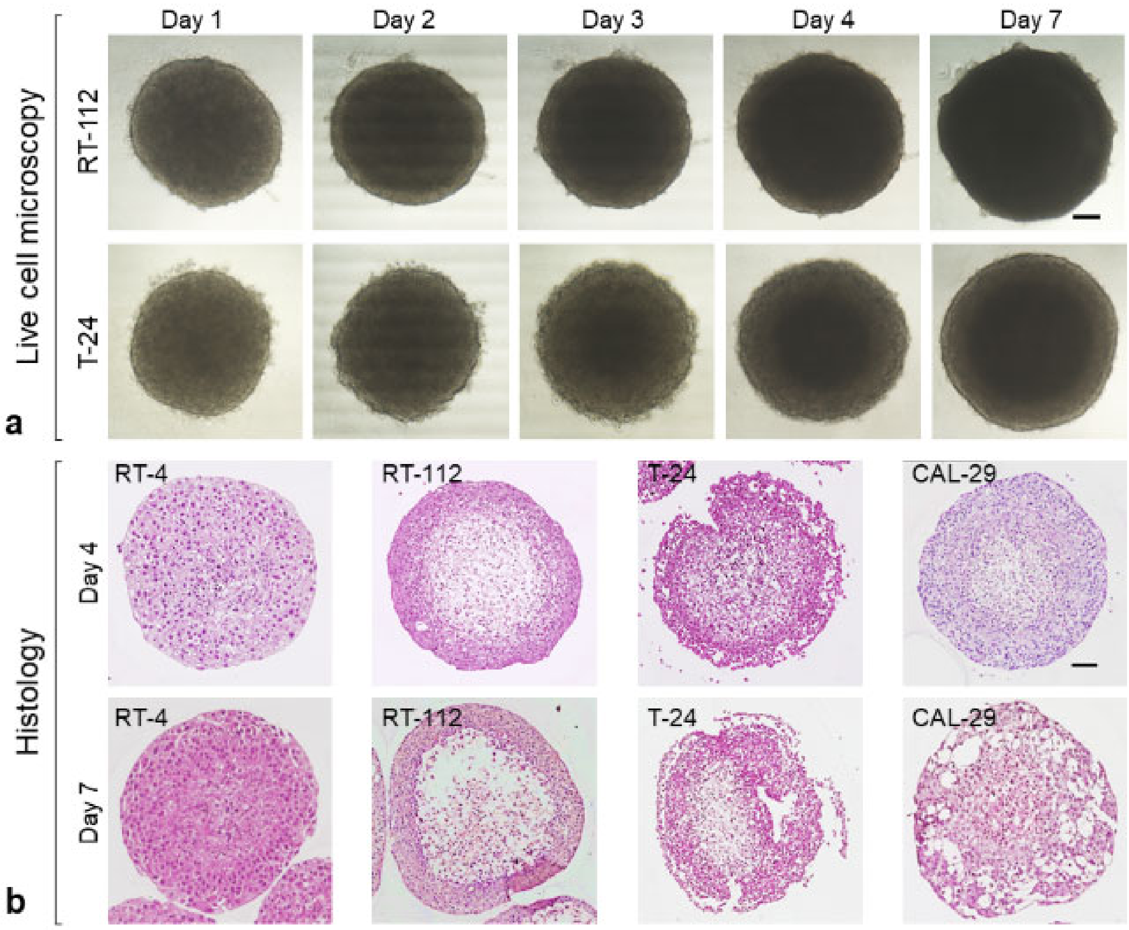

Figure S1. Live cell observation and histology of BCa organoids. BCa cells were co-cultured with hBF and hBSMC in an ULA plate. (a) Development of organoids over a period of up to 7 days. Live cell observation of RT-112 and T-24 organoids by transmitted light. Spheroids reached a size of up to 1 mm in diameter after 1 week. (b) Morphology of human BCa Orgs by HE staining after 4 and 7 days of culture. The morphology of BCa Orgs varied depending on the used BCa cell line; RT-112, T-24 and CAL-29 organoids lost their compact structure after 1 week of culture. Scale bar: 100 µm.
