# Supplementary material for: Cell Line-Based Human Bladder Organoids with Bladder-like Self-Organization—A New Standardized Approach in Bladder Cancer Research
Source: Biomedicines. 2023 Nov 1;11(11):2958. doi: 10.3390/biomedicines11112958 (PMC10669858; doi:10.3390/biomedicines11112958)
Supplement: Supplementary file 1 [file biomedicines-11-02958-s001.zip › Figure S2.pdf]

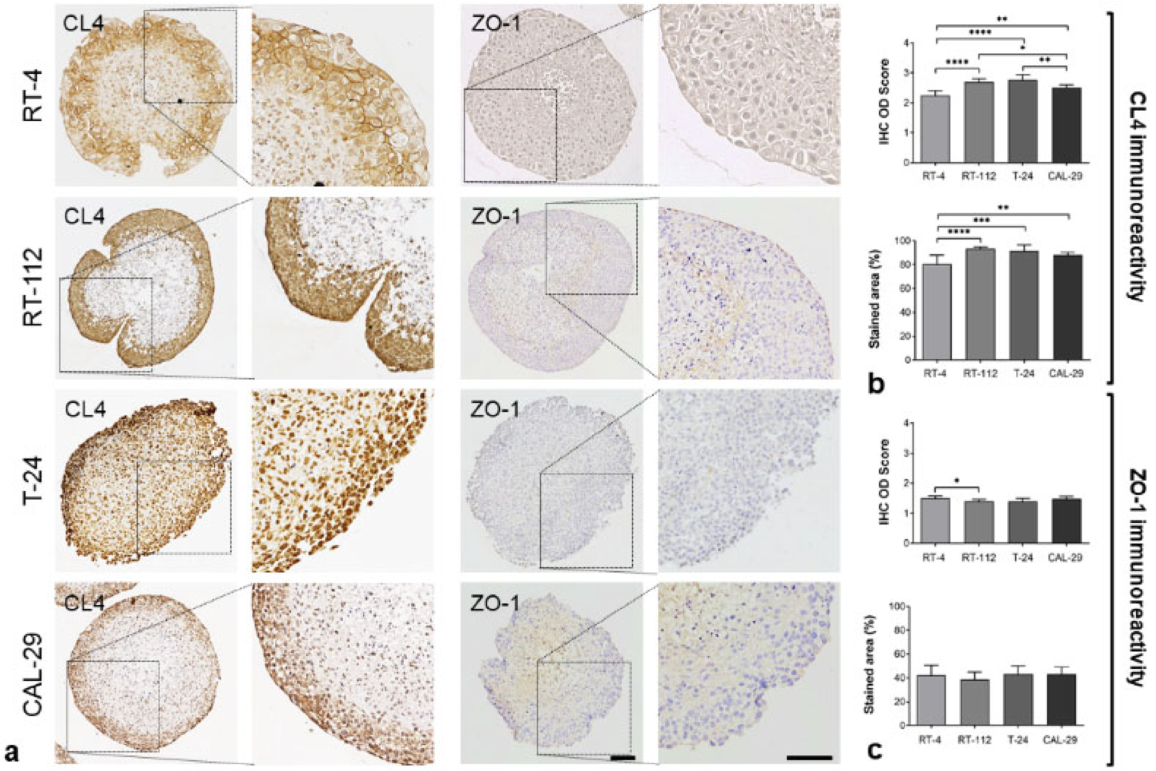

Figure S2: Formation of cell-cell contacts in BCa organoids. Analysis of CL4 and ZO-1 immunoreactivity in BCa cells. (a) Representative images of BCa Orgs immunostained for CL4 and ZO-1 (brown); cell nuclei (blue). CL4 was mainly observed in the urothelial cell layers. ZO-1-IR was not detected. Scale bar: 100 µm. (b) Quantification of CL4 immunoreactivity (IHC OD score; immunohistochemistry optical density score) in the BCa cell layer; CL4-IR was significantly higher in RT-112, T-24 and CAL-29 cells than in RT-4 cells. (c) Quantification of ZO-1 immunoreactivity in the BCa cell layer; all BCa Orgs showed low ZO-1 immunoreactivity; *p < 0.05, **p < 0.01,*** p < 0.001, ****p<0.0001; One-way ANOVA; mean + SD; n=8.
