# Supplementary material for: Cell Line-Based Human Bladder Organoids with Bladder-like Self-Organization—A New Standardized Approach in Bladder Cancer Research
Source: Biomedicines. 2023 Nov 1;11(11):2958. doi: 10.3390/biomedicines11112958 (PMC10669858; doi:10.3390/biomedicines11112958)
Supplement: Supplementary file 1 [file biomedicines-11-02958-s001.zip › Figure S3.pdf]

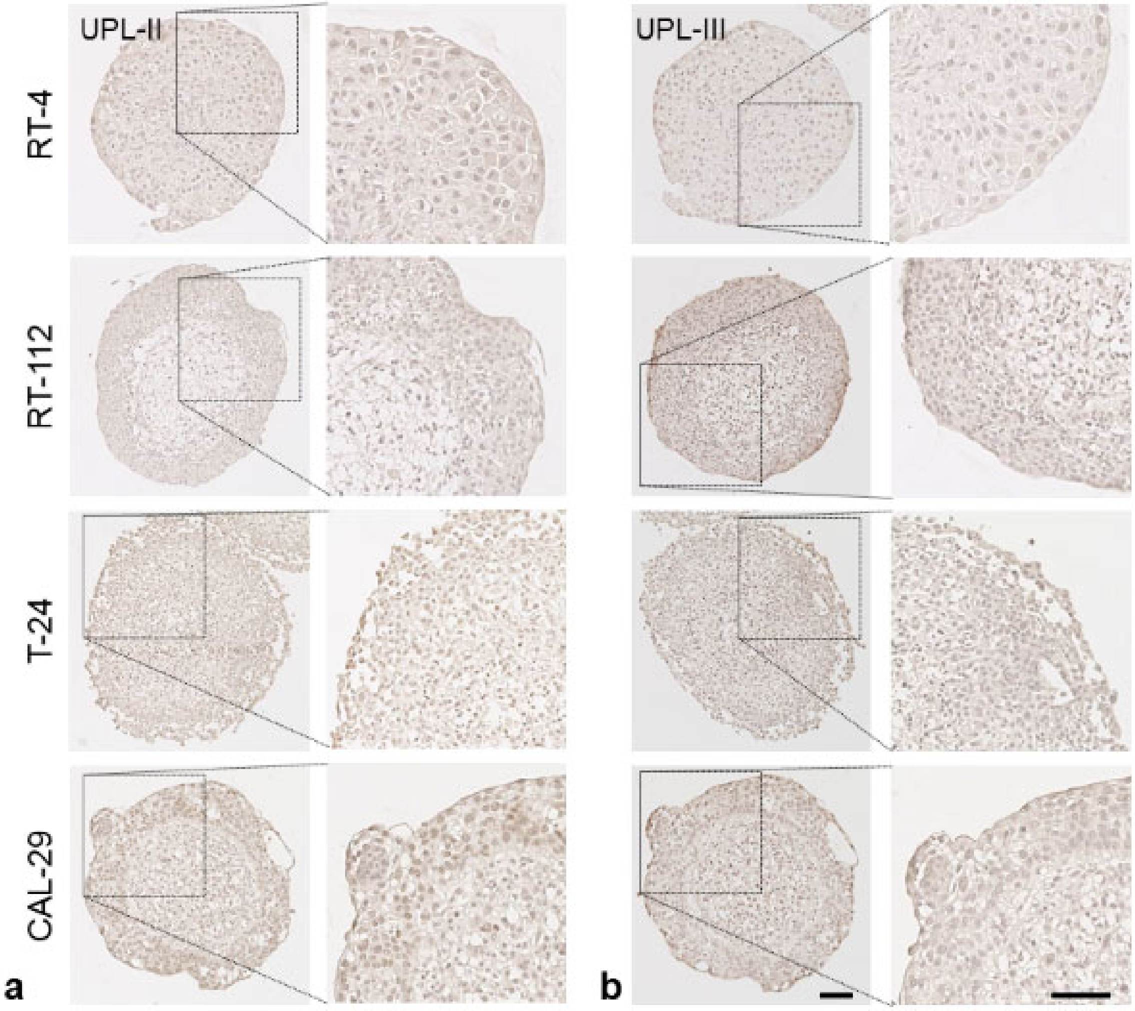

Figure S3: Formation of uroplakin plaques in BCa organoids. Analysis of uroplakin-II (UPL-II) and uroplakin-III (UPL-III) immunoreactivity in BCa cells. (a) Representative images of BCa Orgs immunostained for UPL-II (a, brown) and UPL-III (b, brown); cell nuclei (blue). Uroplakins were almost not existent. Scale bar: 100 µm.
